# Investigating the Sole Olefin-Based Cycle in Small-Cage MCM-35-Catalyzed Methanol-to-Olefins Reactions

**DOI:** 10.3390/molecules29092037

**Published:** 2024-04-28

**Authors:** Zhaohui Liu, Min Mao, Ruixue Yangcheng, Shuang Lv

**Affiliations:** Institute of Advanced Interdisciplinary Studies, School of Chemistry and Chemical Engineering, Chongqing University, Chongqing 400044, China

**Keywords:** methanol to olefins, MCM-35 zeolite, mechanism, olefin-based cycle

## Abstract

Small-pore zeolites catalyze the methanol-to-olefins (MTO) reaction via a dual-cycle mechanism, encompassing both olefin- and aromatic-based cycles. Zeolite topology is crucial in determining both the catalytic pathway and the product selectivity of the MTO reaction. Herein, we investigate the mechanistic influence of MCM-35 zeolite on the MTO process. The structural properties of the as-synthesized MCM-35 catalyst, including its confined cages (6.19 Å), were characterized, confirming them as the catalytic centers. Then, the MTO reactions were systematically performed and investigated over a MCM-35 catalyst. Feeding pure methanol to the reactor yielded minimal MTO activity despite the formation of some aromatic species within the zeolite. The results suggest that the aromatic-based cycle is entirely suppressed in MCM-35, preventing the simultaneous occurrence of the olefin-based cycle. However, cofeeding a small amount of propene in methanol can obviously enhance the methanol conversion under the same studied reaction conditions. Thus, the exclusive operation of the olefin-based cycle in the MTO reaction, independent of the aromatic-based cycle, was demonstrated in MCM-35 zeolite.

## 1. Introduction

Discovered in 1977 by Mobil scientists [1], the methanol-to-hydrocarbons (MTH) reaction has emerged as a crucial non-petroleum synthetic pathway for producing high-demand chemicals, mainly including gasoline (MTG) [2,3], olefins (MTO) [4,5,6,7], propene (MTP) [8,9,10], and aromatics (MTA) [11,12,13]. To elucidate the complex chemistry of MTH and control its product distribution, extensive research has been conducted to reveal its reaction mechanism. Numerous observations and theories have been reported on the MTH mechanisms, particularly focusing on the direct and indirect mechanisms [14,15,16,17,18,19,20,21,22,23]. The direct mechanism regarding the formation of the first C–C bond in the induction time remains controversial and debated, and some recent advanced techniques have proven even new experimental evidence [14,15,16,17,18]. On the contrary, the indirect hydrocarbon pool mechanism, with concrete evidence, is widely accepted in the scientific community [19,20,21,22,23].

In the 1990s, Dahl et al. first proposed the hydrocarbon pool mechanism, positing that methanol feed reacts with the active (CH_2_)_n_ species inside zeolite and generates multiple products and coke species [20,21]. Evolved from the hydrocarbon pool mechanism, a so-called dual-cycle mechanism was demonstrated by the isotopic switching method, which showed that two distinct groups of concurrent active intermediates (olefin and aromatic species) simultaneously work in the reaction to produce complex MTH products [22,23]. The olefin-based cycle determines the products of C_4_–C_7_ aliphatics, while the aromatic-based cycle contributes to the formation of ethene and methane. Another important product, propene, can be released from both cycles and converted in the olefin-based cycle [24,25,26,27]. With more in-depth investigation, it was recognized that feedstock [28,29], reaction condition [30,31,32], and zeolite structure [33,34,35] can influence the relative propagation of the olefin-/aromatic-based cycles and the product distribution as a consequence.

Adjusting zeolite structure is an effective approach to tune catalytic route and product selectivity as well. For example, Khare et al. found that large ZSM-5 crystallite and ZSM-5@SiO_2_ catalysts led to a long intra-crystalline residual time of methylbenzenes, enhancing the aromatic-based cycle to produce a high selectivity of ethene [27]. Yarulina et al. modified ZSM-5 with alkaline earth metals to decrease its acid density and gained more propene than pure ZSM-5, resulting from the suppressed accumulation of aromatics [36]. By promoting the olefin-based catalytic cycle, Zhao et al. achieved a high propene selectivity of 58.3% in the MTP process over a high-Si structured Beta zeolite [37]. We also revealed the effect of the mesoporosity in the Beta catalyst that favored the diffusion of large aromatics and inhibited its relevant catalytic cycle [38]. Notably, ZSM-5 and Beta zeolites have the ability to adjust the propagation of the aromatic-based cycle (i.e., the formation or diffusion of methylbenzenes) because they contain pore sizes comparable to methylbenzenes’ kinetic diameters [27]. Conversely, medium- and large-pore zeolites offer minimal diffusion restriction for smaller olefin intermediates, rendering them challenging to manipulate [39].

Small-pore zeolites, characterized by eight-member-ring cage openings, can restrict aromatic effusion, steering MTH products towards light olefins (MTO) [40,41,42,43]. They are even also capable of confining long-chain aliphatics during the catalytic process, and their cages are readily accommodated with active aromatic intermediates. Most previous studies concur that the aromatic-based cycle is the primary pathway in the MTO reaction [40,41,42,43]. However, many works have recently revealed that the olefin-based species can also serve as scaffolds for the formation of products in the MTO conversion. Via the isotopic tracing approach, Hwang et al. confirmed the coexistence of the olefin-based cycle and aromatic-based cycle in the early time of the MTO process over SAPO-34 [44]. Yang et al. achieved a highest record of 77.3% propene selectivity in one-pass methanol conversion over SAPO-14 zeolite, which was attributed to the olefin-based route dominating the catalytic process [45]. Furthermore, we demonstrated that the propene selectivity can be systematically tuned by adjusting the propagation of the olefin-based cycle in the MTO reaction, determined by the structure of DDR (a type of deea-dodecasil 3R zeolite) catalyst [46]. Based on this knowledge, we hypothesize that tightly constrained zeolite pores could completely inhibit the aromatic-based cycle, allowing solely the olefin-based cycle to facilitate methanol conversion and influence its product distribution.

Bearing that in mind, we chose MCM-35 zeolite as the MTO catalyst in this investigation, which contains a pore topology with a very limiting cage size as small as *o*-xylene. The structural properties of this MTF-type zeolite were confirmed through XRD, NMR, and TEM characterizations. Subsequently, MTO reactions with pure methanol were performed, and the residual species in the spent catalysts were analyzed to assess the catalytic performance. Contrastingly, cofeeding propene with the feedstock was employed to enhance methanol conversion under identical reaction conditions. By analyzing the results, a catalytic mechanism in which the sole olefin-based route working for methanol conversion in the MCM-35-catalyzed MTO reaction was finally approved.

## 2. Results and Discussion

### 2.1. Characterizations of the Catalyst

**Figure 1:**
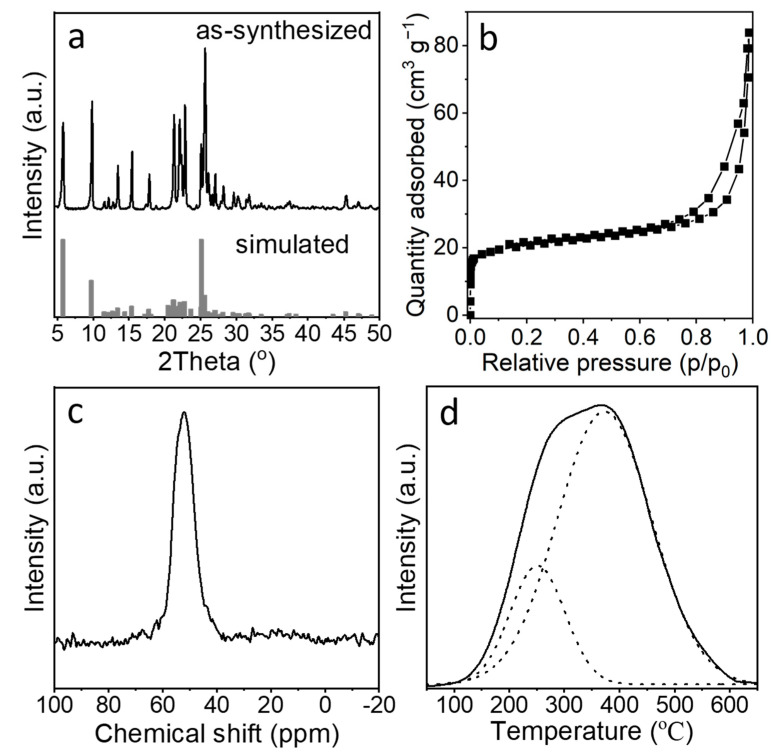
XRD pattern (**a**), N_2_ adsorption−desorption isotherms (**b**), ^27^Al-NMR spectra (**c**), and NH_3_-TPD profile (**d**) of as-synthesized MCM-35 zeolite.

Following a previously reported method (US Patent 4981663) with a slight modification [47], we successfully prepared the MCM-35 (MTF-type) zeolite with a Si/Al ratio of 36 (ICP result). The powder X-ray diffraction pattern (PXRD) indicates that the as-obtained sample is highly crystalline, phase-pure MCM-35 zeolite (Figure 1a). MCM-35 zeolite contains a one-dimensional (1D) interconnecting eight-ring channel system, which shows much smaller surface area and pore volume than other typical small-pore zeolites with large cages. As confirmed by the N_2_ adsorption measurement collected at 77 K (Figure 1b), the as-obtained MCM-35 exhibits a microporous isotherm characteristic, whose BET surface area and micropore volume are 76.4 m^2^/g and 0.025 cm^3^/g, respectively. The N_2_ adsorption at high pressure (p/p_0_ > 0.8) results from the packed pores of the small domain crystals, as confirmed by the TEM images (vide infra).

The distribution of Al sites in the zeolite framework has a significant effect on the activity of zeolite catalysts. ^27^Al (3Q) MAS NMR spectroscopy is a well-developed technique to learn the status of Al sites in zeolite [48]. The result of ^27^Al-NMR measurement (Figure 1c) on the as-synthesized sample exclusively exhibits a signal centered around 55 ppm that corresponds to the tetrahedrally coordinated Al atoms [49], demonstrating the almost complete incorporation of Al sites into zeolite framework. We also carried out the NH_3_-TPD measurement to shed further light on the acidic property of MCM-35. As shown in Figure 1d, the data reveal that MCM-35 contains a small amount of weak acid sites (NH_3_ desorbed at 250 °C) and a large content of strong acid sites (NH_3_ desorbed at 400 °C).

**Figure 2:**
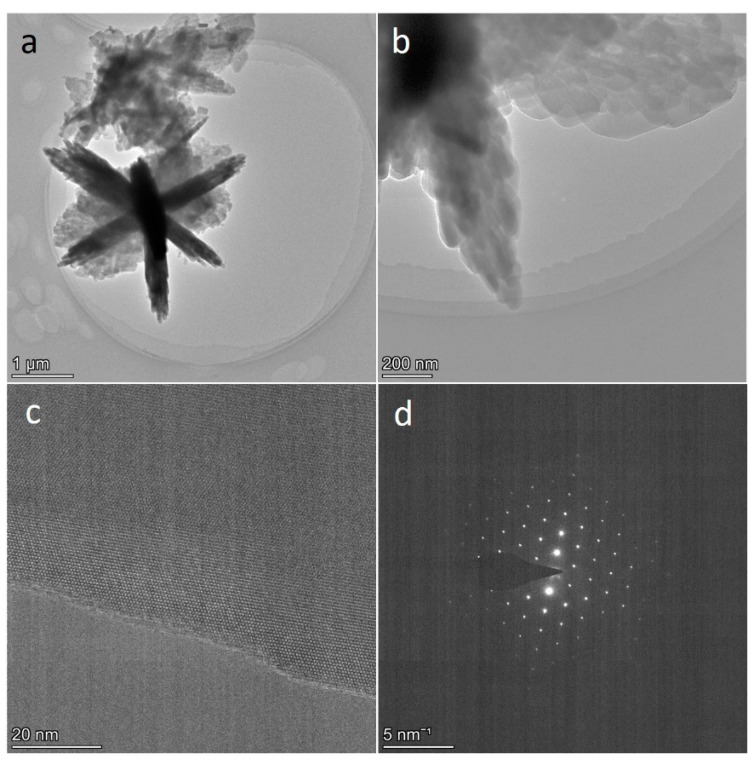
TEM images at low magnification (**a**,**b**), high-resolution TEM images (**c**), and SEAD pattern (**d**) of MCM-35 zeolite.

To learn the structural details of the obtained MCM-35 zeolite, its micrographs were then imaged by SEM and TEM. As clearly shown in the SEM images in Figure S1, the micro-sized MCM-35 particles are assembled by platelike crystals. TEM experiments were then performed to visually determine the shape of the sample. As shown in Figure 2a,b, the plate-shaped particles of MCM-35 zeolite are over 2 µm in diameter, and they are composited with the domains of about 100 nm in size, as detected in the TEM images at low magnification. These images show that the whole MCM-35 particles are hierarchical structures with small domains packed together. Previously, it was documented that MCM-35 zeolite usually presents a platelet morphology, with a preferred orientation of 1D channels normal to the [010] dimension of the crystals [50]. When we zoomed in on this crystalline face of MCM-35 zeolite with a higher TEM magnification, its micropores could be clearly observed, as shown in Figure 2c, with a rather high crystallinity shown by the SEAD pattern in Figure 2d. These characterizations indicate that the catalytically active sites are mainly present in the micropores of MCM-35 crystals.

### 2.2. Catalytic Reactions

The operation of the MTO process is generally determined by the cage size of small-pore zeolites. Via operando UV−vis spectroscopy, Goetze et al. revealed the differences in the MTO performances of CHA, DDR and LEV zeolites, in terms of active intermediates, product selectivities, and deactivation behaviors [42]. Recently, Davis and coworkers have established a relationship between cage size and product distribution in the MTO reactions carried out over 14 different cage-type topologies. Notably, it was found that there was no MTO activity for 1D MCM-35 zeolite, and the main product was dimethyl ether (DME) [51]. To deepen our understanding of this issue, we compared the cage sizes of MCM-35 with those of RHO and SAPO-34 (Figure S2). RHO and SAPO-34, the MTO active zeolites, have cage sizes of 10.43 Å and 7.45 Å, respectively. However, the cage size of MCM-35 is only 6.19 Å [52]. Previously, it was commonly known that the aromatic-based species were active intermediates in the MTO reaction due to the confinement effect of small-pore zeolite. Therefore, it was thought that the cage size of MCM-35 was too narrow for methylbenzene intermediates. However, our previous results showed that the olefin-based catalytic intermediates can also function within the MTO process and influence the product distribution [46], which could potentially serve as the working species in MCM-35 cages as well. With that assumption in mind, we carried out the MTO reaction over MCM-35 and also co-fed active olefins (propene and ethene) with methanol to learn their effects on catalytic behaviors of MCM-35 catalyst.

**Figure 3:**
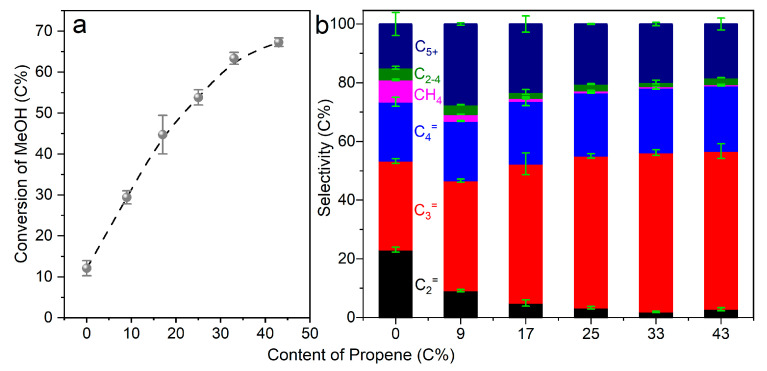
Conversion (**a**) and product distribution (**b**) of MTO on MCM-35 zeolites with varied amounts of propene co-fed in methanol. The MTO reactions were performed at 400 °C and analyzed at 10 min time-on-stream.

The reactions were carried out in a quartz fixed-bed reactor at 400 °C under the pressure of MeOH at 5.2 kPa. The weight hourly space velocity (WHSV) was set to 2.4 g_MeOH_ g_Cat_^−1^ h^−1^. We analyzed the effluent at a steady state (10 min time-on-stream) of the MTO reaction over MCM-35 and found a limited methanol conversion of only 12.1% (Figure 3a). A high selectivity of light olefins exceeding 70% was obtained without any aromatics detected in the effluent, exhibiting a typical MTO product distribution (Figure 3b). This result clearly shows that the small-pore MCM-35 zeolite has a rather weak MTO activity, which is consistent with the previous report [51]. Notably, when we co-fed only 3 mol% propene with methanol in the feedstock, the conversion of methanol increased to 29.4%, meaning that the propene served as an active intermediate in the MTO reaction (Figure 3a). When deeply analyzing the product distribution, we found that the selectivities towards thermal cracking products methane and ethene decreased from 7.5 C% to 2.3 C% and 23.1 C% to 9.2 C%, respectively. The selectivity of the olefin-based long-chain aliphatics (C_5+_) increased from 14.8 C% to 27.5 C% (Figure 3b). Further, when we added more propene in the methanol, the conversion of methanol increased to 67.3%. However, the selectivity of C_5+_ aliphatics was decreased again from 3 mol% propene cofeeding gas flow to 10 mol% propene cofeeding gas flow, while the amount of propene increased simultaneously. We think the reason should be that the methylation of olefins dominated the catalytic process when the methanol amount was high in the feed, while the cracking of long-chain aliphatics happened to a great extent when methanol was largely consumed in the reactor. However, cofeeding more propene (10–20 mol%) did not improve the catalytic behaviors of MCM-35 under the studied reaction conditions, which was limited by its catalytic capacity at 400 °C (Figure 3). When we performed the similar reactions at a higher temperature of 500 °C, an almost complete methanol conversion was achieved with a 14 mol% propene co-fed in methanol (Figure S3).

The widely accepted dual-cycle concept provides a mechanistic basis for understanding the MTO process. It is worth noting that the aromatic-based cycle contributes to the formation of ethene and methane, while the olefin-based cycle leads to the production of long-chain aliphatics. To correlate the effect of propene with the catalytic cycles, we performed a reaction of pure propene with MCM-35 catalyst under 400 °C and found no conversion occurred in the reaction with just a tiny amount of ethene existing, which might come from the slight cracking of propene (Figure S4). These experimental results demonstrate that the methylation of propene dominates the conversion of propene and methanol in the reactions studied above. We also studied the influence of cofeeding ethene on the methanol conversion and found a smaller promotion occurred than that of cofeeding propene. A 43.6% conversion of methanol was achieved by cofeeding 29 mol% ethene in methanol (Figure S5), showing the presented methylation process of ethene with methanol as well. Previously, Hill et al. reported that the rate of propene methylation is at least an order of magnitude faster than that of ethene [53]. However, they did not consider small-pore zeolites under their investigation. However, the diffusion rate of ethene is much faster than propene in the small-pore zeolites [54]. Therefore, the apparent rate of ethene methylation was of similar magnitude to that of propene, and it also promoted the methanol conversion under our studied reaction conditions in this work.

Further, we also monitored the lasting effect of co-fed propene by pausing it at 5–10 min. We compared three different reactions, which were R1 with the feedstock of pure methanol at 0–10 min, R2 with the feedstock of 86 mol% methanol + 14 mol% propene at 0–10 min, and R3 with the feedstock of 86 mol% methanol + 14 mol% propene at 0–5 min and switched to pure methanol at 5–10 min (Figure S6). The results show that the methanol conversion decreased from 63.4 in R2 to 35.2% in R3, with the consumption of propene and the formation of C_5+_ aliphatics in the effluent. In a typical MTO catalyst (such as SAPO-34), the accumulation of methylbenzenes and the propagation of the aromatic-based cycle readily occurred in the cages and worked at the MTO process. However, methylbenzenes, especially the heavy-branched ones, cannot serve as the active intermediates in the small cages of MCM-35 zeolite, while olefins can act as scaffolds for methanol conversion. In contrast to methylbenzenes, olefins could be consumed on the active sites or easily diffuse out from the crystals. Therefore, a continued cofeeding operation of propene is needed to keep the lasting methanol conversion, as proven in our results mentioned above.

### 2.3. Analysis of the Residual Organic Species in the Used Catalysts

Next, we investigated the remaining organic species formed in MCM-35 cages to obtain more information about the catalytic performances discussed above. We extracted the residual organic species in the spent MCM-35 samples after the MTO reactions performed at 400 °C and a WHSV of 2.4 g_MeOH_ g_Cat_^−1^ h^−1^. After the 10 min time-on-stream reaction, the heater was stopped, and the quartz-tube reactor was quickly cooled down under flowing air gas. When the reactor was completely cooled down to room temperature, samples were taken out and dissolved with an HF solution, and then the organic species were extracted with CH_2_Cl_2_ from the water phase [55,56,57]. These remaining organic species in the spent MCM-35 cages were detected via GC-MS.

**Figure 4:**
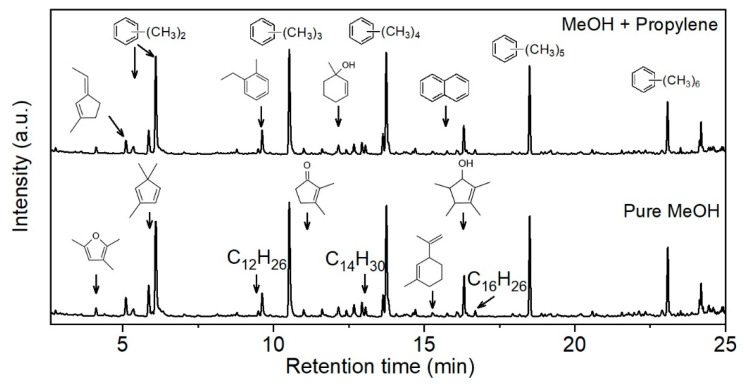
GC analysis of the remaining organic species extracted from spent MCM-35 samples. Reaction conditions: at 400 °C, the feedstock is pure MeOH (**bottom**), the feedstock is 80 mol% MeOH + 20 mol% propylene (**top**), and the time-on-stream is 10 min.

As shown in Figure 4, reactions with the feedstock of pure methanol and the feedstock of methanol (80 mol%) + propene (20 mol%) led to similar residual organic species in MCM-35 cages. The methylbenzenes are the main compounds retained in catalyst, including small xylenes and large penta-/hexa-methylbenzenes. The cage size of MCM-35 is about 6.19 Å, which is similar to the kinetic diameter of *o*-xylene (6.2 Å) but much smaller than that of hexamethylbenzene (7.2 Å) [58]. The formation of heavy methylbenzenes or even naphthalene could happen through cell expansion of zeolite [59] or the cage-passing growth mechanism [60]. However, the dealkylation of branched methylbenzenes cannot follow the paring or the side-chain routes to produce light olefins due to the confined space in MCM-35 cages. These results could explain why MCM-35 has no MTO activity with pure methanol feed. In addition, cyclic alkenes and long-chain aliphatics, which are considered intermediates in the olefin-based catalytic cycle, are presented but also inactive in MCM-35 cages. Many deactivating cyclopentenone derivatives also exist in catalysts, as found in our previous work [61].

Due to the formation of these deactivating species, as well as the formed coke in the small channels of MCM-35 zeolite, MCM-35 also has a very short lifetime in the MTO reaction, which is similar to the small-pore zeolites. We also carried out the long-term test of the MTO reaction that was performed at 500 °C and 20% propene co-fed in methanol. As shown in Figure S7, the methanol conversion decreased sharply from 97.3 mol% at 10 min to 23.0 mol% at 50 min. When analyzing the product selectivity, we found that the ethene quickly decreased, and the C_5+_ increased accordingly. The results demonstrated that the cracking reactions were favored at the beginning of the reaction due to the high number of catalytic sites, while the methylation of olefins was favored at the end of the MTO reaction because of the fade of the catalytic sites in MCM-35 zeolite.

**Scheme 1:**
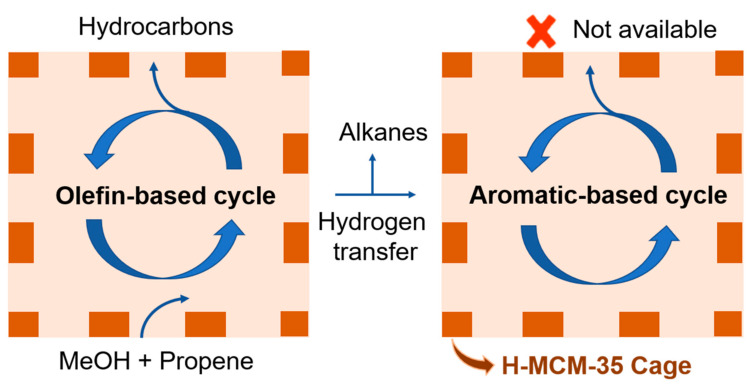
Mechanistic illustration of the catalytic cycles for the MTO reaction over MCM-35 zeolite.

With the experimental observations, we tried to explain the mechanistic basis for the MCM-35-catalyzed MTO reaction. The aromatic-based species were generally known as the active intermediates in the small-pore zeolite, which can be formed in MCM-35 zeolite as well (Figure 4). However, the aromatic-based catalytic cycle does not work in its cages, where we think the dealkylation process of branched methylbenzenes is prohibited by the confined space (Scheme 1). In contrast, the branched methylbenzenes can work for the MTO process when it is catalyzed by other small-pore zeolites with larger-sized cages (e.g., SAPO-34 and RHO) due to the fact that there is enough working space for these intermediates [62]. In our MCM-35 zeolite studied here, the olefin species can serve as the intermediates for methanol conversion inside of the 6.19 Å-sized cages of MCM-35. Inevitably, the active olefins either transform into aromatics or diffuse out of the catalyst. Therefore, the propagation of the olefin-based cycle is also suppressed when the MTO feedstock is pure methanol on MCM-35 zeolite. We believe that this evidence could explain why previous works found that MCM-35 had no MTO activity [51,52]. More importantly, we revealed that cofeeding active ethene/propene can largely promote methanol conversion, demonstrating that the sole olefin-based catalytic cycle can work for the MTO reaction on MCM-35 zeolite, as shown in Scheme 1.

## 3. Material and Methods

### 3.1. Synthesis of Zeolite

MCM-35 was synthesized according to a previously reported method in the US Patent of 4,981,663 with minor modifications [47]. Specifically, 0.6 g of Al_2_(SO_4_)_3_·18H_2_O (Shanghai Aladdin Biochemical Technology Co., Shanghai, China) was first dissolved into 25 mL H_2_O, and then a solution of 1.14 g of 45 wt% KOH (Shanghai Aladdin Biochemical Technology Co., Shanghai, China) was added to it. After stirring for 10 min, 4.25 g of Cabosil M-5 (Shanghai King Chemical Co., Ltd., Shanghai, China) was subsequently added into the above solution and thoroughly stirred for 0.5 h. Finally, 2.1 g of hexamethyleneimine (Shanghai Aladdin Biochemical Technology Co., Ltd, Shanghai, China) was added, and the gel was put into an autoclave for crystallization at 175 °C for 12 days with a rotational speed of 40 rpm. The products were thus collected and washed with H_2_O at least 3 times. After being air-dried at 100 °C, the samples were finally calcined at 550 °C for 6 h. In order to obtain the H-form zeolite, the as-prepared zeolite sample was ion-exchanged with a 0.5 M NH_4_NO_3_ solution overnight (using 20 mL per gram of zeolite) 3 times at 70 °C. The powder was then recovered, washed with H_2_O, dried in air, and then calcined at 550 °C in air for 4 h.

### 3.2. Catalyst Characterizations

Scanning electron microscopy (SEM) image was taken on a FEI Helios 5. Transmission electron microscopy (TEM) images and high-resolution TEM images were obtained on an FEI Talos 200S operated at 200 kV. The XRD pattern was obtained on a PANalytical Instrument with Cu Kα radiation (λ = 0.1542 nm). An N_2_ adsorption–desorption isotherm was obtained on a MicrotracBEL apparatus for the sample first degassed at 120 °C for 12 h before the analysis. The total surface area was calculated based on the Brunauer−Emmett−Teller (BET) equation and the micropore volume was evaluated using the *t*-plot method. An inductively coupled plasma optical emission spectrometer (ICP-OES) was performed on an iCAP 6300 Duo apparatus from Thermo Scientific company. Magic-angle spinning (MAS) ^27^Al single-pulse nuclear magnetic resonance (NMR) spectra were recorded on a Bruker Advance 400 M NMR spectrometer operating at a magnetic field of 9.4 T. The hydrated samples were packed into a 4.0 mm ZrO_2_ rotor. Spectra were recorded at a resonance frequency of 104.2 MHz, a spinning rate of 5 kHz, a pulse length of 0.6 μs, and a recycle delay of 0.5 s for about 1000 scans. Temperature-programmed desorption (TPD) measurements using NH_3_ as the probe molecule were performed on a Micromeritics AutoChem II 2950 apparatus. Before measurements, 0.10 g sample was pretreated in He gas (25 mL/min) for 2 h at 500 °C and then cooled to 100 °C. Next, the sample was exposed to a mixed gas (10 mol% NH_3_ and 90 mol% He) flow of 20 mL/min for 0.5 h to ensure the sufficient adsorption of NH_3_. Prior to desorption, the sample was flushed in He gas for 3 h. Subsequently, NH_3_ desorption was performed in the range of 50–650 °C at a heating rate of 10 °C/min under a He flow of 20 mL/min.

### 3.3. The MTO Reactions

The MTO conversion was carried out in a quartz fixed-bed reactor (6 mm in OD; 2 mm in wall thickness) packed with 100 mg H-form MCM-5 zeolite catalyst diluted with ~500 mg quartz sand. The catalyst was first activated in air flow (20 mL/min) at 500 °C for 1 h, following which the catalyst bed was cooled down to a certain reaction temperature, and the air gas was changed to a N_2_ gas flow (50 mL/min) mixed with a 5 µL/min methanol liquid controlled by a syringe pump. Different amounts of propene were co-fed in the reaction gas to change the catalytic behaviors regarding the methanol conversion and product selectivity. The reactions were performed under atmospheric pressure, and the products were analyzed using online gas chromatography (GC 9720Plus, FULI INSTRUMENTS) with a flame ionization detector (FID) equipped with Agilent HP-PLOT/Q column (30 m × 0.53 mm × 40 μm); dimethyl ether (DME) was not considered as a product for the calculation.

### 3.4. Analysis of the Residual Organic Species in the Used Catalysts

The soluble organic species that remained in spent catalysts were extracted with a normally applied approach [55,56,57]. In detail, the spent catalyst was transferred to a capped Teflon vial and dissolved in 10 mL of 24% HF for 1 h. Then, 3.0 mL CH_2_Cl_2_ was used to extract the organic species from the H_2_O phase. Subsequently, the organic CH_2_Cl_2_ phase was separated from the mixture and measured in a GC–MS setup (GCMS-TQ8040, SHIMADZU Corporation, Kyoto, Japan) equipped with an Agilent HP-5 column (30 m × 0.32 mm × 0.25 μm).

## 4. Conclusions

The significant role of the olefin-based cycle plays in the MTO reaction has been largely overlooked in previous studies. Drawing on our understanding of olefin intermediates’ effects, we selected MCM-35 zeolite as the MTO catalyst for this study. Contrary to the extensively studied MTO catalysts, MCM-35 zeolite has ultrasmall cages that restrict the diffusion of methylbenzenes and hinder their catalytic function, thereby inhibiting and excluding the aromatic-based catalytic cycle. However, in this catalyst, the olefin-based cycle’s propagation is also limited by the consumption or diffusion of active olefin species, for which the methanol conversion is merely 12.1% when the feedstock is pure methanol at 400 °C and a WHSV of 2.4 g_MeOH_ g_Cat_^−1^ h^−1^. The results demonstrate that these two catalytic cycles are tightly connected together, and the products from the aromatic-based cycle are needed for the proceeding of the olefin-based cycle. To enhance the olefin-based catalytic route, co-feeding a specific amount of active olefin with methanol enabled us to achieve an optimal methanol conversion of 67.3%, with 20 mol% propene co-fed in methanol under identical conditions. A further analysis of the catalyst’s residual species revealed that the present methylbenzenes are ineffective for methanol conversion, and the aromatic-based cycle is entirely precluded within the MCM-35 cages. More importantly, our findings demonstrate that the olefin-based catalytic cycle solely operates for the MTO reaction on MCM-35 zeolite when cofeeding active olefins. This study broadens our understanding of the olefin-based cycle in small-pore zeolite-catalyzed MTO reactions.

## Data Availability

The original contributions presented in the study are included in the article and Supplementary Material, further inquiries can be directed to the corresponding authors.

## Supplementary Materials

The following supporting information can be downloaded at https://www.mdpi.com/article/10.3390/molecules29092037/s1: Figure S1. SEM images of MCM-35. Figure S2. Cage sizes of RHO, SAPO-34 and MCM-35 zeolites. Figure S3. Conversion (a) and product distribution (b) of MTO on MCM-35 zeolites with varied amount of propene cofed in methanol. MTO reactions were performed at 500 °C and analyzed at 10 min time-on-stream. Figure S4. Reaction of propene with MCM-35 catalyst. The flow rate of propene is 2 mL/min and the catalyst amount is 100 mg. Figure S5. Conversion (a) and product distribution (b) of MTO on MCM-35 zeolites with varied amount of ethene cofed in methanol. MTO reactions were performed at 400 °C and analyzed at 10 min time-on-stream. Figure S6. Conversion (a) and product distribution (b) of MTO on MCM-35 zeolites. Reaction conditions: R1 with the feedstock of pure methanol (0–10 min), R2 with the feedstock of 86 mol% methanol + 14 mol% propene (0–10 min), R3 with the feedstock of 86 mol% methanol + 14 mol% propene (0–5 min) and switched to pure methanol (5–10 min), MTO reactions were performed at 400 °C and analyzed at 10 min time-on-stream. Figure S7. The evolution of methanol conversion and product selectivity in MCM-35 catalyzed MTO reaction. MTO reactions were performed at 500 °C and 20% propene was co-fed in methanol.
