# Supplementary material for: Investigating the Sole Olefin-Based Cycle in Small-Cage MCM-35-Catalyzed Methanol-to-Olefins Reactions
Source: Molecules. 2024 Apr 28;29(9):2037. doi: 10.3390/molecules29092037 (PMC11085503; doi:10.3390/molecules29092037)
Supplement: Supplementary file 1 [file molecules-29-02037-s001.zip › molecules-2935841-supplementary.pdf]

Supporting Information

Investigating the Sole Olefin-Based Cycle in Small-Cage MCM-35-Catalyzed Methanol-to-Olefins Reactions

Zhaohui Liu*, Min Mao, Ruixue Yangcheng, Shuang Lv

Institute of Advanced Interdisciplinary Studies, School of Chemistry and Chemical Engineering, Chongqing University, Chongqing 400044, China

*Corresponding author. Email: zhaohui.liu@cqu.edu.cn

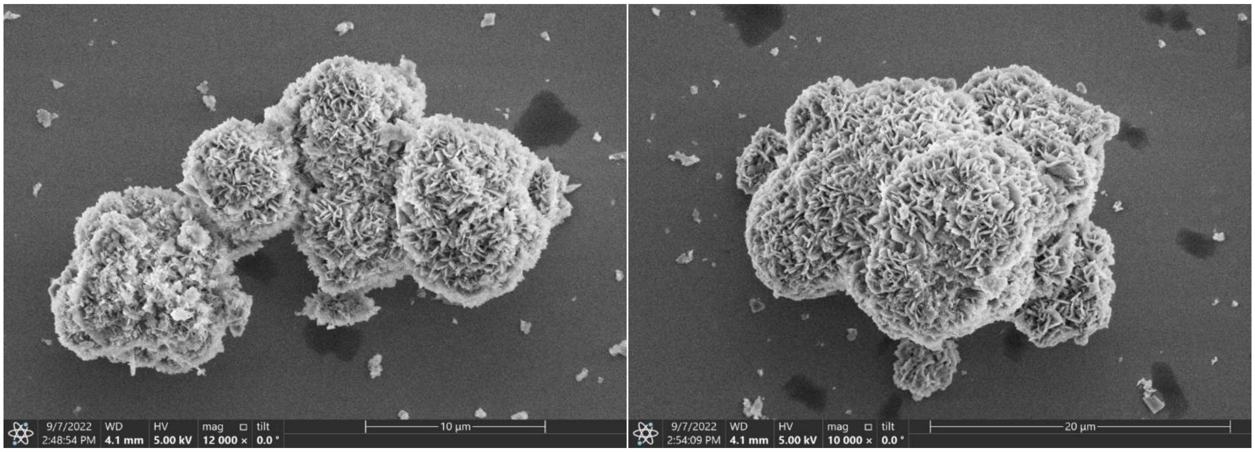

Figure S1. SEM images of MCM-35.

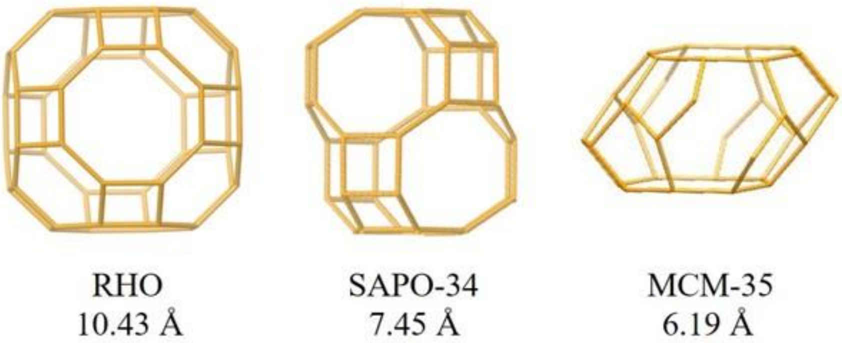

Figure S2. Cage sizes of RHO, SAPO-34 and MCM-35 zeolites.

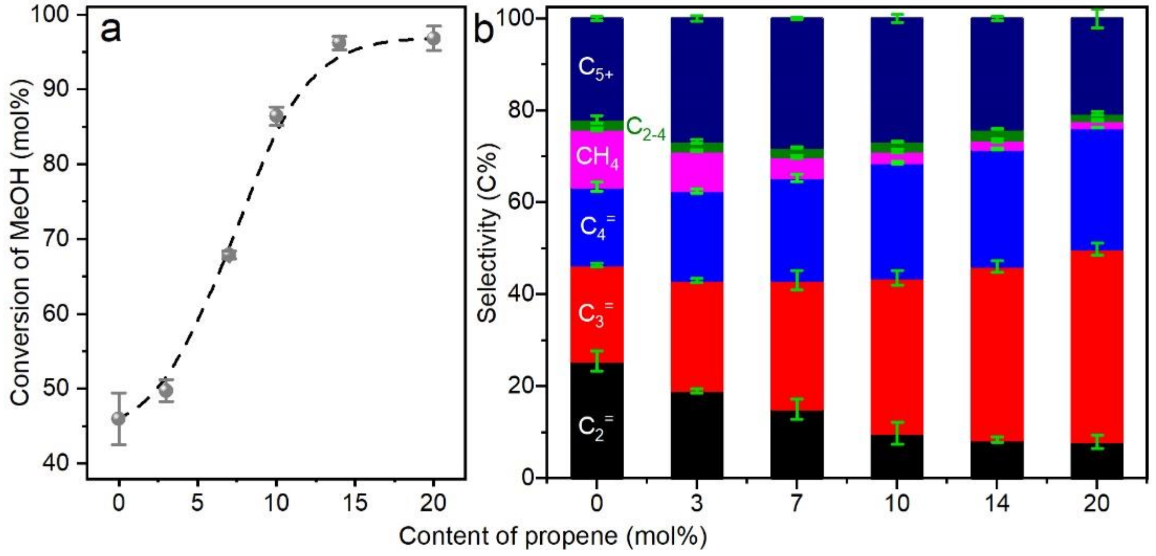

Figure S3. Conversion (a) and product distribution (b) of MTO on MCM-35 zeolites with varied amount of propene cofed in methanol. MTO reactions were performed at 500 °C and analyzed at 10 min time-on-stream.

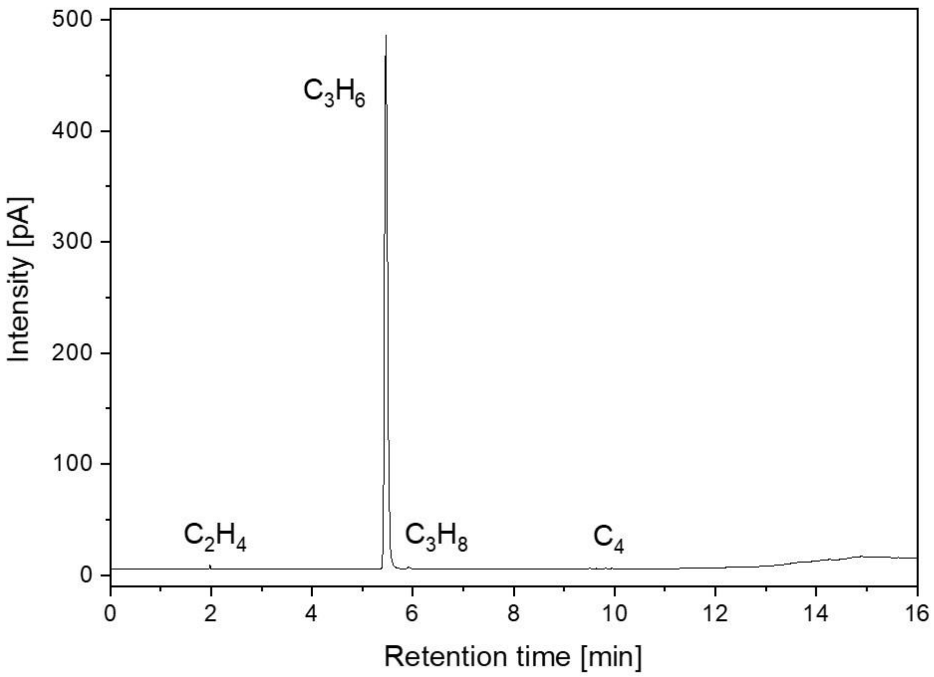

Figure S4. Reaction of propene with MCM-35 catalyst. The flow rate of propene is 2 mL/min and the catalyst amount is 100 mg.

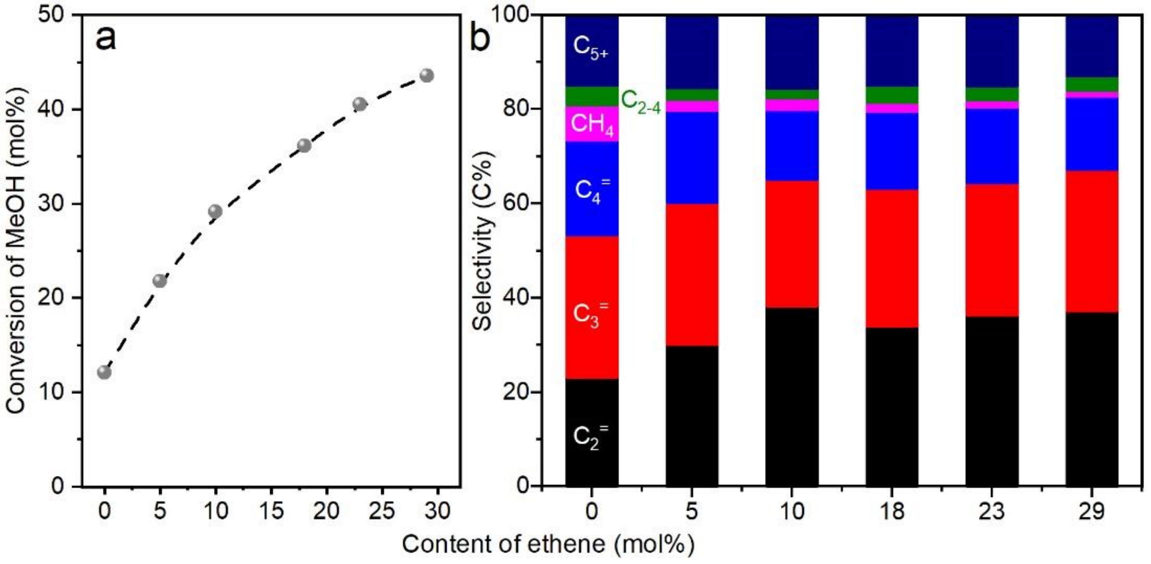

Figure S5. Conversion (a) and product distribution (b) of MTO on MCM-35 zeolites with varied amount of ethene cofed in methanol. MTO reactions were performed at 400 ℃ and analyzed at 10 min time-on-stream.

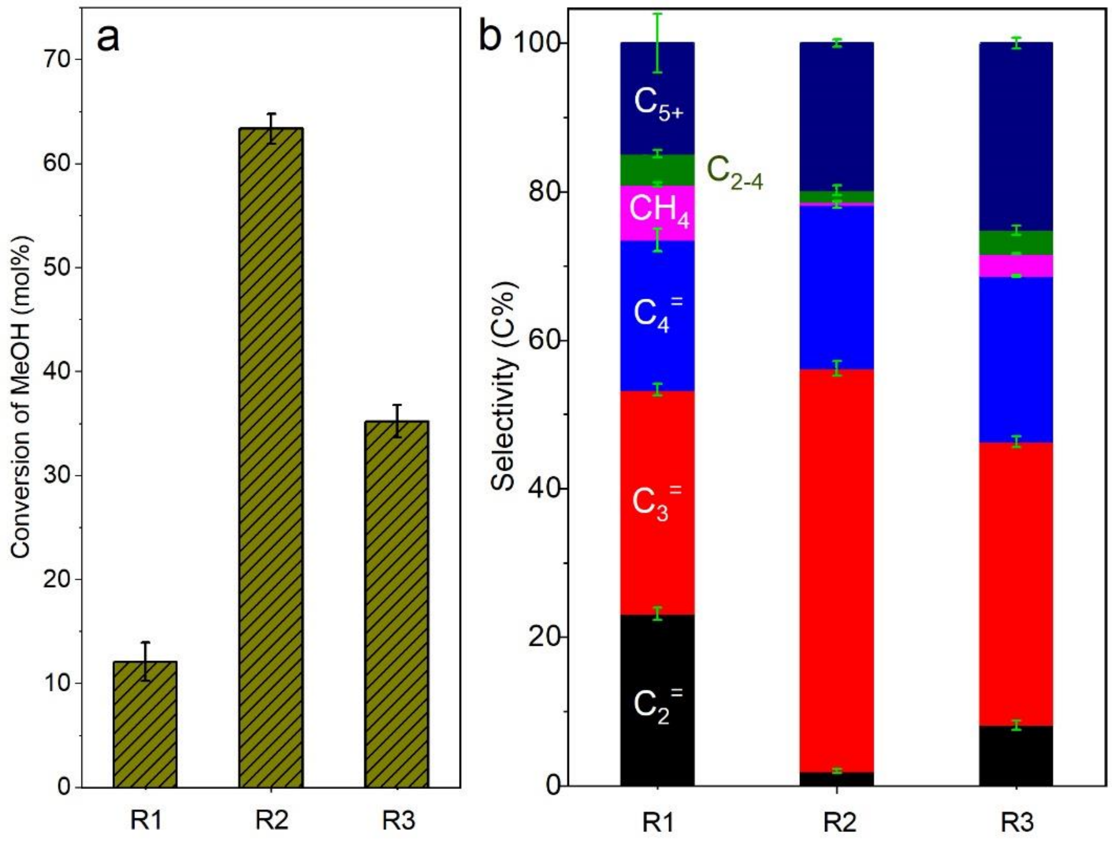

Figure S6. Conversion (a) and product distribution (b) of MTO on MCM-35 zeolites. Reaction conditions: R1 with the feedstock of pure methanol (0-10 min), R2 with the feedstock of 86

mol% methanol + 14 mol% propene (0-10 min), R3 with the feedstock of 86 mol% methanol + 14 mol% propene (0-5 min) and switched to pure methanol (5-10 min), MTO reactions were performed at 400 °C and analyzed at 10 min time-on-stream.

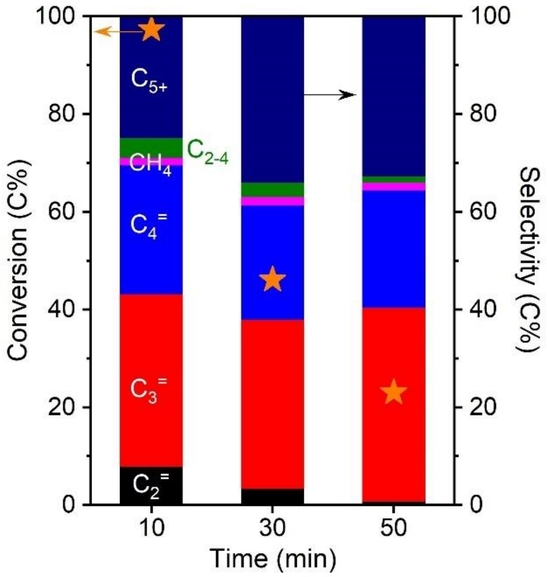

Figure S7. The evolution of methanol conversion and product selectivity in MCM-35 catalyzed MTO reaction. MTO reactions were performed at 500 °C and 20% propene was co-fed in methanol.
